# The machine learning‐based model for lateral lymph node metastasis of thyroid medullary carcinoma improved the prediction ability of occult metastasis

**DOI:** 10.1002/cam4.7155

**Published:** 2024-05-29

**Authors:** Xiwei Zhang, Xiaohui Zhao, Lichao Jin, Qianqian Guo, Minghui Wei, Zhengjiang Li, Lijuan Niu, Zhiqiang Liu, Changming An

**Affiliations:** ^1^ Department of Head and Neck Surgical Oncology, National Cancer Center/National Clinical Research Center for Cancer/Cancer Hospital Chinese Academy of Medical Sciences and Peking Union Medical College Beijing China; ^2^ Department of Ultrasound Qilu Hospital of Shandong University Jinan China; ^3^ Department of Head and Neck Surgical Oncology National Cancer Center/National Clinical Research Center for Cancer/Cancer Hospital & Shenzhen Hospital, Chinese Academy of Medical Sciences and Peking Union Medical College; ^4^ Department of Ultrasound National Cancer Center/National Clinical Research Center for Cancer/Cancer Hospital, Chinese Academy of Medical Sciences and Peking Union Medical College Beijing China; ^5^ Department of Radiation Oncology National Cancer Center/National Clinical Research Center for Cancer/Cancer Hospital, Chinese Academy of Medical Sciences and Peking Union Medical College Beijing China

**Keywords:** lateral lymph node metastasis, machine learning, medullary thyroid carcinoma, mobile health applications, prophylactic neck dissection, TI‐RADS

## Abstract

**Background:**

For medullary thyroid carcinoma (MTC) with no positive findings in the lateral neck before surgery, whether prophylactic lateral neck dissection (LND) is needed remains controversial. A better way to predict occult metastasis in the lateral neck is needed.

**Methods:**

From January 2010 to January 2022, patients who were diagnosed with MTC and underwent primary surgery at our hospital were retrospectively reviewed. We collected the patients' baseline characteristics, surgical procedure, and rescored the ultrasound images of the primary lesions using American College of Radiology (ACR) Thyroid Imaging, Reporting and Data System (TI‐RADS). Regularized logistic regression, 5‐fold cross‐validation and decision curve analysis was applied for lateral lymph node metastasis (LLNM) model's development and validation. Then, we tested the predictive ability of the LLNM model for occult LLNM in cN_0−1a_ patients.

**Results:**

A total of 218 patients were enrolled. Five baseline characteristics and two TI‐RADS features were identified as high‐risk factors for LLNM: gender, baseline calcitonin (Ctn), tumor size, multifocality, and central lymph node (CLN) status, as well as TI‐RADS margin and level. A LLNM model was developed and showed a good discrimination with 5‐fold cross‐validation mean area under curve (AUC) = 0.92 ± 0.03 in the test dataset. Among cN_0−1a_ patients, our LLNM model achieved an AUC of 0.91 (95% CI, 0.88–0.94) for predicting occult LLNM, which was significantly higher than the AUCs of baseline Ctn (0.83) and CLN status (0.64).

**Conclusions:**

We developed a LLNM prediction model for MTC using machine learning based on clinical baseline characteristics and TI‐RADS. Our model can predict occult LLNM for cN_0−1a_ patients more accurately, then benefit the decision of prophylactic LND.

## 1 INTRODUCTION

Medullary thyroid carcinoma (MTC) is a relatively rare neuroendocrine tumor (NET) that originates from the parafollicular C cells. It has an incidence of less than 1 per 100,000 population and accounts for only 1%–2% of all thyroid malignancies.^1^, ^2^ The standard treatment for MTC is total thyroidectomy and central neck dissection (ND). For patients with clinically or radiologically detected lateral lymph node metastasis (LLNM) before surgery, therapeutic lateral neck dissection should be performed.^2^, ^3^, ^4^ For MTC patients without evidence of LLNM, whether prophylactic lateral neck dissection (LND) should be performed is still controversial.

Some research suggest that central lymph node (CLN) metastasis^5^ and high preoperative basal calcitonin (Ctn)^6^ are high‐risk factors for LLNM, and recommend to decide whether to perform further prophylactic lateral ND based on this. However, there is no widespread consensus in clinical practice, nor is it recommended by most guidelines. Due to the higher malignancy of MTC compared to differentiated thyroid carcinoma (DTC), patients with MTC without preoperative evidence of LLND could face the dilemma of undertreatment or overtreatment with LND. Therefore, how to accurately predict the presence of occult LLNM in cN_0−1a_ patients before surgery is an urgent problem that needs to be solved.

In the past decade, machine learning has been widely applied in various fields of medical research, and the advent of GPT‐4 has even ignited the global desire for artificial intelligence. Machine learning algorithms, such as deep learning, artificial neural networks, traditional classification, and probabilistic models under supervised learning,^7^ have been employed by some studies to develop prediction models for thyroid tumors, such as to judge benignancy or malignancy, distinguish pathological types, predict lymphnode metastasis, BRAFV600E mutations and treatment outcomes, etc.^8^, ^9^, ^10^, ^11^, ^12^

Previous studies have developed some models to predict lymph node metastasis or prognosis for MTC, but they mostly involved limited cases.^13^, ^14^, ^15^, ^16^ Our study is based on a single center and incorporates TI‐RADS scores along with clinical baseline data to achieve a better prediction outcome. Moreover, we evaluated the performance of our model in detecting occult LLNM by testing it among cN_0−1a_ patients, and compared it with basal Ctn and CLN status to demonstrate its clinical applicability.

## 2 MATERIALS AND METHODS

### 2.1 Patient enrollment and data collection

**FIGURE 1:**
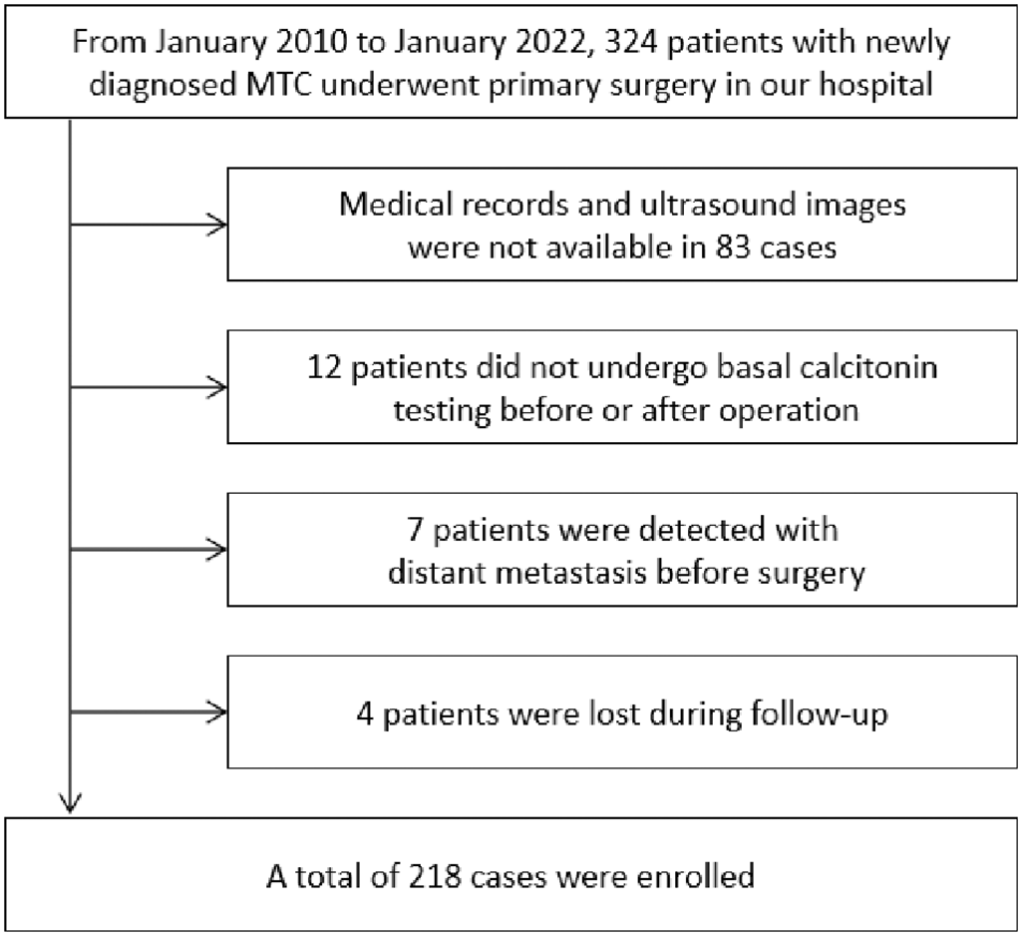
Flow chart of patient enrollment.

From January 2010 to January 2022, 324 patients who underwent initial surgery and pathologically confirmed medullary thyroid cancer in our hospital were reviewed. Our exclusion criteria were (1) previous clinical records and ultrasound images were unavailable or of poor quality, (2) pre‐ and postsurgery basal Ctn tests were unavailable, (3) distant metastasis was detected before surgery, and (4) biochemically uncured patients were lost to follow‐up and could not determine the recurrence of lateral neck. A total of 218 cases were included in our research. The flow chart of patient enrollment is shown in Figure 1.

This study has been registered and approved by the Ethics Committee of the Cancer Hospital, Chinese Academy of Medical Sciences. (Approval number: NCC2021C‐431). Considering that it is a retrospective study, signing informed consent was judged exempted.

We reviewed the medical records and extracted the patient's clinical baseline characteristics, namely: age, gender, family history (based on RET germline gene screening, judging whether the patient is sporadic or inherited MTC), preoperative basal Ctn, tumor size (tumor length, if multiple lesions, the larger one is regarded as the main lesion), multifocality, CLN status. The criteria of radiographically positive lymph nodes was determined based on the presence of enlarged size, cystic changes, rounded shape, eccentric cortical, widening, decreased echogenicity, loss of fatty hilus, presence of calcifications, and increased intranodal vascularity.^17^, ^18^ Based on the American College of Radiology (ACR) Thyroid Imaging, Reporting and Data System (TI‐RADS), the ultrasound features were recorded with data for nodule composition, echogenicity, shape, size, margins, and echogenic foci. The features for each patient were independently evaluated and recorded in a blinded manner by two radiologists (Qianqian Guo and Lijuan Niu), with 5 and 30 years of experience in thyroid nodules diagnosis. When a disagreement occurred between the two reviewers during valuation, a joint review was performed, and consensus data were used for further statistical analysis.

### 2.2 Surgery and outcome assessment

According to the preoperative ultrasound and neck CT images results, 126 patients were diagnosed as cN_0_, 15 patients as cN_1a_, and 77 patients as cN_1b_ in the group. Except for one with inherited MTC who had prophylactic total thyroidectomy, all patients underwent total thyroidectomy and central compartment dissection. Additionally, 38 cases in the cN_0_ group and 9 cases in the cN_1a_ group underwent prophylactic LND. The specific surgical procedures and treatment outcomes are shown in Table 1.

**TABLE 1:** The specific surgical procedures and treatment outcomes.

	*n*	Total TD	Total TD + CND	Total TD + Prophylactic LND	Total TD + Therapeutic LND	pN_1b_ n.(%)	Biochemical cure *n* (%)
cN_0_	126	1	86	38	—	23 (18.3)	100 (79.4)
cN_1a_	15	—	6	9	—	9 (60.0)	7 (46.7)
cN_1b_	77	—	—	—	77	71 (92.2)	14 (18.2)

Abbreviations: TD, thyroidectomy; CND, central neck dissection; LND, lateral neck dissection.

According to the postoperative pathological results, 23, 9, and 71 patients in the cN_0_, cN_1a_, and cN_1b_ groups were found to have metastasis in the lateral neck, respectively, totaling 103 cases. The biochemical cure rates of the three groups were respectively 79.4%, 46.7%, and 18.2%. For patients who underwent LND, those who were pathologically confirmed LNM were defined as positive; otherwise, they were defined as negative. For patients who did not undergo LND, if the postoperative Ctn was lower than normal (biochemically cure), it was defined as negative. If biochemical cure was not achieved, the patients were followed up by outpatient review records and telephone calls. A total of five patients were found to have lateral lymphnode recurrence in follow‐up.

### 2.3 Feature selection

MTCs with and without LLNM were compared based on patient clinical baseline characteristics and TI‐RADS score. The features used in this study include numeric features and categorical features. The numeric features were age, tumor size, and basal Ctn. The categorical features were gender, multifocality, family history (inherited or sporadic MTC), CLN status, TI‐RADS items (composition, echogenicity, shape, margin, echogenic foci), and TI‐RADS level. The Chi‐squared test and Student's *t*‐test were used to determine whether there was any statistical difference in categorical features and numeric features. The features with *p*‐value <0.05 (gender, multifocality, central compartment LN, TI‐RADS edge and TI‐RADS level, tumor size, and basal Ctn) were selected for further study. The statistical analysis was performed by using Python software (version 3.8).

### 2.4 Model development

In this study, we developed a regularized logistic regression model for LLNM prediction. The numeric features were standard scaled, and the categorical features were one‐hot encoded via ‘OneHotEncoder’ in the preprocessing. Then, the hyperparameters for LLNM prediction model are optimized by cross‐validated exhaustive grid search over the search space. The strategy to select the best hyperparameters is to select the most accurate prediction in the training set. For more details of the model development, please refer to Note S1.

We utilized 5‐fold cross‐validation to split our study population into training and testing datasets to reduce bias and avoid overfitting in this study. The screened 218 MTC patients were randomly divided into five equal parts, each of which was called a fold. The LLNM prediction model was trained on 4 folds of the patients at a time, and then tested on the remaining fold patients. This process was repeated five times so that each fold of the data has a chance to become the independent test dataset.

### 2.5 Model evaluation

The performance of the LLNM prediction model was evaluated by quantitative indexes including the area under the receiver operating characteristic (ROC) curve (AUC), accuracy (ACC), sensitivity/recall, specificity, positive predictive value/precision, negative predictive value, Matthew's correlation coefficient, and F1 score (F1), which are described in Note S2. The decision curve analysis was used to test the clinical usefulness of the regularized logistic regression model in LLNM prediction. The details can refer to Note S3.

To test the clinical utility of the LLNM model, we evaluated its ability to predict occult LLNMs in cN_0−1a_ patients, which is 141 cases in total. We also examined the predictive ability of Basal Ctn and CLN status, and compared that with the LLNM model. The evaluation method is the same as described above.

## 3 RESULTS

### 3.1 Patient characteristics and selection of significant features

In this study, 107 cases were diagnosed with LLNM and 111 cases without. There were five clinical baseline characteristics and two TI‐RADS items associated with LLNM in patients, details are shown in Table 2. The risk factors for LLNM in baseline characteristics included: male, high calcitoninemia, large tumor, multifocal lesions, and CLN metastasis. Tumor margin and overall level of TI‐RADS significant difference were seen between the groups.

**TABLE 2:** Baseline characteristics and TI‐RADS scores of the 218 patients in medullary thyroid carcinoma with and without lateral cervical lymph node metastases.

Characteristics		Without LLNM (*n* = 111)	With LLNM (*n* = 107)	*p*‐Value
Age (years)^a^		49 (13–78)	50 (21–75)	0.995
Gender^b^				
Male		39 (35.1)	63 (58.9)	<0.001
Female		72 (64.9)	44 (41.1)	
Family history				
Sporadic MTC		98 (88.3)	96 (89.7)	0.728
Inherited MTC		13 (11.7)	11 (10.3)	
Basal Ctn (pg/mL)^a^		221.0 (6.6–8192.0)	2000.0 (12.4–74428.0)	<0.001
Tumor size (mm)^a^		12.0 (2.6–49.0)	23.0 (4.0–68.0)	<0.001
Number of lesions^b^				
Unifocal		91 (82.0)	66 (61.7)	<0.001
Multifocal		20 (18.0)	41 (38.3)	
CLN status^c^				
Positive		107 (96.4)	46 (43.0)	<0.001
Negative		4 (3.6)	61 (57.0)	
ACR TI‐RADS^d^				
Composition^b^				
Cystic or spongiform	0	0 (0.0)	0 (0.0)	0.222
Mixed cystic and nodule	1	11 (9.9)	5 (4.7)	
Solid	2	100 (90.1)	102 (95.3)	
Echogenicity^b^				
Anechoic	0	0 (0.0)	0 (0.0)	0.491
Hyperechoic or isoechoic	1	3 (2.7)	2 (1.9)	
Hypoechoic	2	89 (80.2)	80 (74.8)	
Very hypoechoic	3	19 (17.1)	25 (23.4)	
Shape^b^				
Wider‐than‐tall	0	105 (94.6)	97 (90.7)	0.392
Taller‐than‐wide	3	6 (5.4)	10 (9.3)	
Margin^b^				
Smooth or Ill‐defined	0	22 (19.8)	7 (6.5)	<0.001
Lobulated or Irregular	2	87 (78.4)	69 (64.5)	
Extra‐thyroidal extension	3	2 (1.8)	31 (29.0)	
Echogenic foci^b^				
None or large comet‐tail artifacts	0	49 (44.1)	38 (35.5)	0.393
Macrocalcifications	1	41 (36.9)	48 (44.9)	
Peripheral (rim) calcifications	2	0 (0.0)	0 (0.0)	
Punctate echogenic foci	3	21 (18.9)	21 (19.6)	
TI‐RADS level^b^				
TR1	0	0 (0)	0 (0)	0.016
TR2	2	1 (0.9)	0 (0.0)	
TR3	3	5 (4.6)	0 (0.0)	
TR4	4–6	35 (31.5)	22 (20.6)	
TR5	7+	70 (63.1)	85 (79.4)	

Abbreviations: ACR, American College of Radiology; CLN, central lymphnode; Ctn, calcitonin; LLNM, lateral lymphnode metastasis; MTC, medullary thyroid cancer; TI‐RADS, thyroid imaging, reporting and data system.

^a^ Data are presented as medians with ranges in parentheses.

^b^ Data in parentheses are percentages.

^c^ Preoperative ultrasound considering central lymph node metastasis or not.

^d^ The number on the right represents the score of each item in ACR TI‐RADS.

### 3.2 Prediction performance on cross‐validation cohort

**FIGURE 2:**
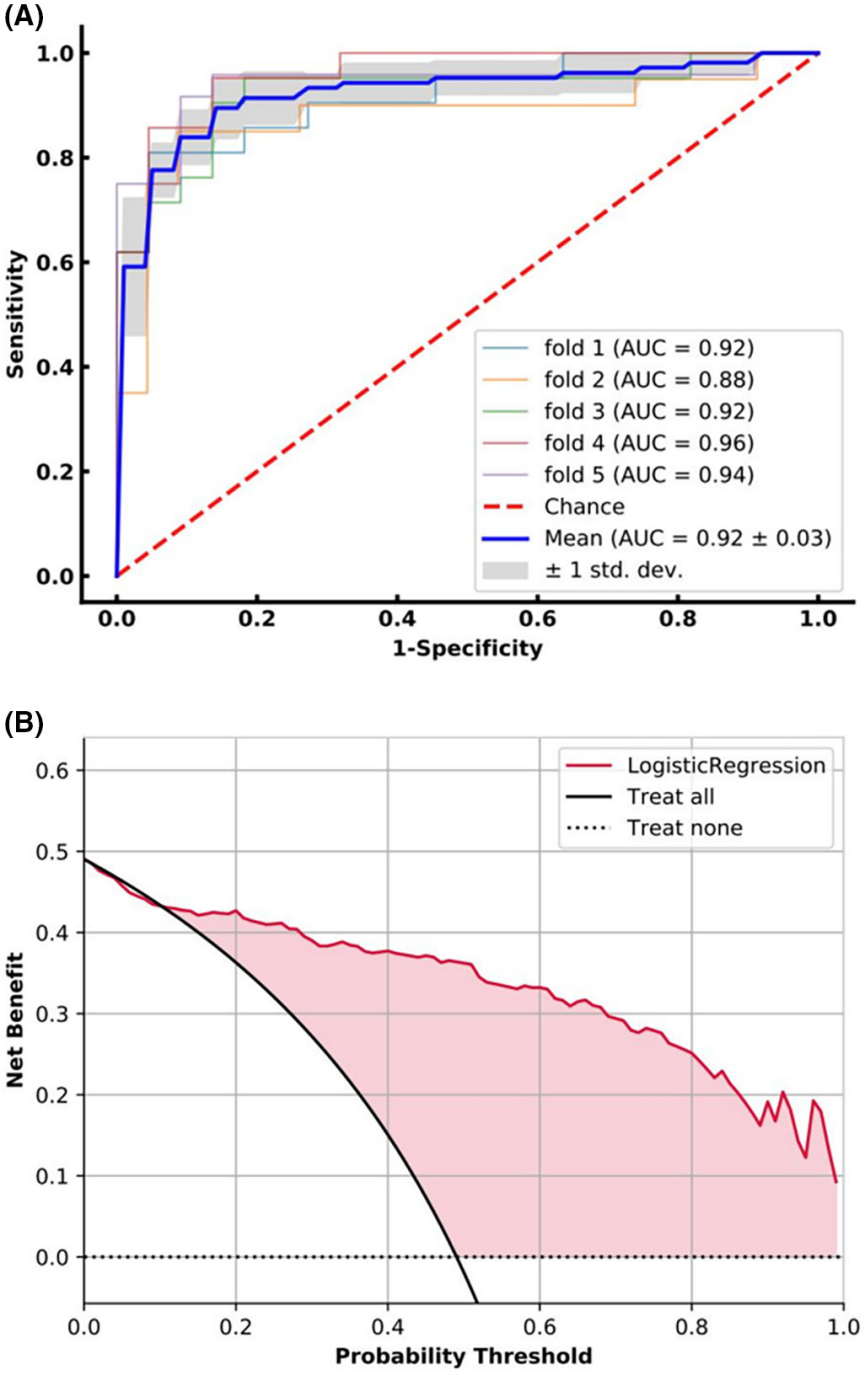
Receiver operating characteristic (ROC) curve and decision analysis curve of lateral lymph node metastasis (LLNM) prediction model on 5‐fold cross‐validation testing datasets. (A) ROC curves of the LLNM prediction results on testing datasets, the area under curve of the LLNM prediction model on average is 0.92 ± 0.03 with one standard deviation. (B) Decision analysis curve of the LLNM prediction model, which shows that if the threshold probability ranged from 12% to 99%, using the LLNM prediction model can gain more benefit than treat all or none patients.

**FIGURE 3:**
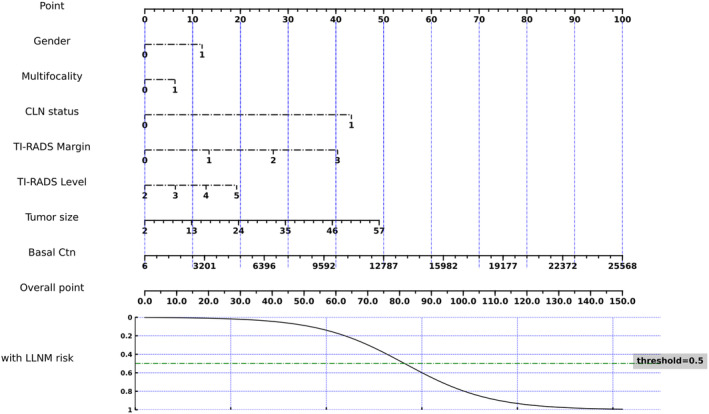
The nomogram of the Fold 4 among the five modelings. Points were assigned for gender, multifocality, central lymph node status, Thyroid Imaging, Reporting and Data System (TI‐RADS) margin, TI‐RADS level, tumor size, and basal calcitonin by drawing a line upward from the corresponding values to the “Points” line. The sum of these seven points, plotted on the “overall points” line, corresponds to predictions of LLNM risk, with the threshold = 0.5.

Because of lacking an external validation set, we utilized 5‐fold cross‐validation to split our study population into training and testing datasets. The averaged quantitative indexes of LLNM prediction status for each clinical feature type on testing datasets was summarized in Table 3. Figure 3 shows the nomogram of the Fold 4 among the five modelings, which roughly displays the weights of each clinical feature. The total score of the seven features corresponds to the risk of LLNM. From the experimental results (Table 4), the AUCs of the LLNM prediction model on the five cross‐validation testing sets were 0.92, 0.88, 0.92, 0.96, and 0.94, respectively, and averaged to be 0.92 ± 0.03 with one standard deviation. Figure 2A illustrated the ROC curves of the LLNM prediction results on the cross‐validation cohort. The corresponding quantitative indexes of the LLNM prediction model were summarized in Table S1

**TABLE 3:** Averaged quantitative indexes of LLNM prediction status by different types of clinical features on 5‐fold cross‐validation testing dataset.

Feature type	AUC	ACC	SEN	SPE	PPV	NPV	MCC	F1
Gender	0.61	0.62	0.58	0.65	0.61	0.63	0.23	0.60
Multifocality	0.60	0.61	0.39	0.82	0.68	0.58	0.23	0.49
CLN status	0.77	0.77	0.57	0.96	0.94	0.70	0.58	0.71
Tumor size	0.75	0.69	0.63	0.75	0.71	0.68	0.38	0.66
Basal Ctn	0.87	0.74	0.59	0.89	0.85	0.70	0.51	0.69
TI‐RADS Margin	0.67	0.58	0.84	0.34	0.63	0.80	0.28	0.65
TI‐RADS Level	0.59	0.58	0.80	0.37	0.56	0.65	0.18	0.65
All	0.92	0.87	0.82	0.92	0.91	0.85	0.75	0.86

Abbreviations: ACC, accuracy; AUC, area under curve; CLN, central lymph node; Ctn, calcitonin; F1, F1 score; LLNM, lateral lymph node metastasis; MCC, Matthew's correlation coefficient; NPV, negative predictive value; PPV, positive predictive value; SEN, sensitivity, SPE, specificity; TI‐RADS, Thyroid Imaging, Reporting and Data System.

**TABLE 4:** Quantitative indexes of LLNM prediction model on 5‐fold cross‐validation testing dataset.

	AUC	ACC	SEN	SPE	PPV	NPV	MCC	F1
Fold 1	0.92	0.84	0.71	0.95	0.94	0.78	0.69	0.81
Fold 2	0.88	0.88	0.85	0.91	0.89	0.88	0.77	0.87
Fold 3	0.92	0.88	0.90	0.86	0.86	0.90	0.77	0.88
Fold 4	0.96	0.91	0.86	0.95	0.95	0.88	0.82	0.90
Fold 5	0.94	0.85	0.79	0.91	0.90	0.80	0.70	0.84
Average	0.92	0.87	0.82	0.92	0.91	0.85	0.75	0.86

Abbreviations: ACC, accuracy; AUC, area under curve; F1, F1 score; LLNM, lateral lymph node metastasis; MCC, Matthew's correlation coefficient; NPV, negative predictive value; PPV, positive predictive value; SEN, sensitivity, SPE, specificity.

**FIGURE 4:**
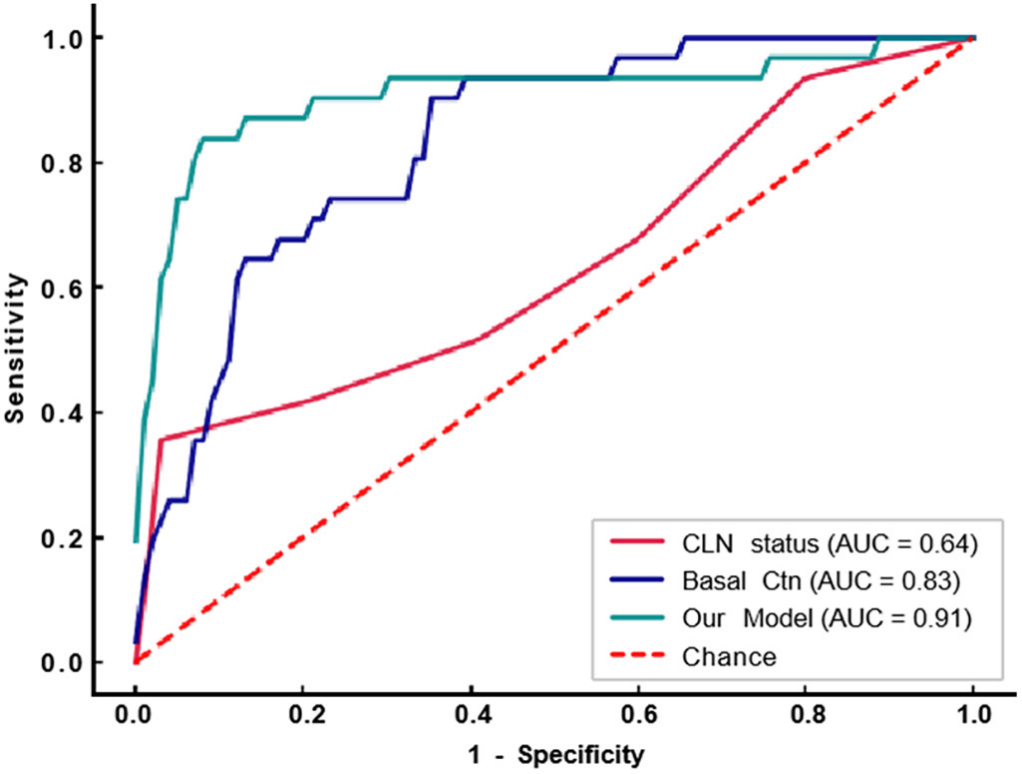
Receiver operating characteristic curves for occult lateral lymph node metastasis (LLNM) prediction by our LLNM model, basal calcitonin and central lymph node status in cN_0−1a_ patients.

Figure 2B illustrated the averaged decision curve of the LLNM prediction model on 5‐fold cross‐validation testing dataset. The decision curves showed that if the threshold probability ranged from 12% to 99%, using the LLNM prediction model can gain more benefit than treat all or none patients.

### 3.3 Clinical utility

Our model demonstrated good predictive ability for occult LLNM in 141 patients with cN_0−1a_, achieving an AUC of 0.91 (95% CI, 0.83–0.98), which was superior to basal Ctn's 0.83 (95% CI, 0.76–0.91) and CLN status's 0.64 (95% CI, 0.52–0.75).(see detail in Table 5) Figure 4 shows the ROC curve. The Delong test showed a significant difference among the 3 models (Table 6). Our model also had a higher ACC of 0.9, compared to basal Ctn and CLN status's 0.79 and 0.83, respectively. The specific parameter index is shown in Table S2. This indicates that our model can better determine whether to perform prophylactic LND for patients with negative founding lateral neck before surgery.

**TABLE 5:** Quantitative indexes of our LLNM model, basal Ctn and CLN status on cN_0−1a_ patients.

	AUC	ACC	SEN	SPE	PPV	NPV	MCC	F1
CLN status	0.64	0.83	0.35	0.97	0.79	0.84	0.45	0.49
Basal Ctn	0.83	0.79	0.45	0.9	0.56	0.85	0.38	0.5
Our Model	0.91	0.9	0.81	0.93	0.78	0.94	0.73	0.79

Abbreviations: ACC, accuracy; AUC, area under curve; CLN, central lymph node; Ctn, calcitonin; F1, F1 score; LLNM, lateral lymph node metastasis; MCC, Matthew's correlation coefficient; NPV, negative predictive value; PPV, positive predictive value; SEN, sensitivity, SPE, specificity.

**TABLE 6:** DeLong test for ROC curves of our LLNM model, basal Ctn and CLN status in 141 cN_0−1a_ patients.

	*P*
CLN status & Basal Ctn	0.006
CLN status & Our model	<0.001
Basal Ctn & Our model	0.028

Abbreviations: CLN, central lymph node; Ctn, calcitonin; LLNM, lateral lymph node.

## 4 DISCUSSION

Compared to differentiated thyroid carcinoma, MTC is more aggressive and has a poorer prognosis, especially when lymph node metastasis occurs.^19^ Thus, the treatment strategy for MTC should focus on assessing the cervical lymph nodes. The standard surgery is total thyroidectomy plus CLN dissection. Controversy exists regarding prophylactic lateral neck dissection for patients without preoperative evidence of lateral neck involvement. We developed a machine learning model for LLNM prediction using clinical and ultrasound data of 218 patients. The model demonstrated a robust performance with a 5‐fold AUC of 0.92 ± 0.03. Furthermore, the model exhibited superior predictive ACC for occult LLNM compared to basal Ctn and CLN status (AUC 0.91 vs. 0.83 vs. 0.64). By providing a more precise risk assessment, we can avoid both overtreatment and undertreatment, tailoring the treatment strategy to each individual's unique needs.

There have been some studies trying to build models to predict LLNM of MTC, based on a limited sample size.^14^, ^15^, ^16^ Zhou et al.^15^ developed a nomogram based on the five clinical characteristics of 35 patients from single center, and their prediction model had discrimination with a C‐index of 0.825. We contend that the sample size of 35 cases, relying solely on clinical features for refining predictive characteristics, raises reliability concerns. Similar to our article, Luo et al.^16^ developed a prediction model for cervical lymph node metastasis (CLNM) by applying clinical and ultrasonographic data of 74 patients with MTC. Their model had a high discrimination with an AUC of 0.919. However, their model could not tell the location of CLNM in the central or lateral neck. Therefore, their model had limited help for solving the current dilemma of whether to perform prophylactic LND for MTC.

Some studies have suggested that prophylactic LND for MTC patients with no evidence of LLNM does not improve prognosis. The study from MD Anderson^20^ analyzed the data of 66 sporadic MTC patients and found no significant difference in biochemical cure rate, local recurrence rate, and other outcomes between the elective lateral neck dissection group and the observation group. However, in this study, the prophylactic LND group had significantly higher Ctn levels and relatively higher T‐stage (although not statistically significant) than the non‐LND group, which proved that prophylactic LND improved the prognosis of patients with more severe conditions. In another study from MSKCC,^21^ it was observed that in patients with Ctn >200 pg/mL, the recurrence rate at 10 years was lower in the prophylactic LND group than in the non‐LND group, 21% versus 30%, although there was no statistical difference since the limited cases. From these two studies, prophylactic LND can bring benefits, but the benefits are not significant. Therefore, if a better method is used to predict the lateral neck metastasis, the value of prophylactic LND will be more evident.

We chose to use the ACR TI‐RADS mainly because it is a more comprehensive and objective system for describing the ultrasound features of the primary lesion, and it has a wide application base worldwide. Therefore, we believe that it can reflect the primary lesion in a comprehensive way, and also facilitate the dissemination of our model in other medical centers. The ACR TI‐RADS scale is a system for scoring thyroid nodules based on their ultrasound features, proposed by the American College of Radiology in 2017.^22^ By assigning points to five categories of ultrasound images and TI‐RADS level, the scale was used to determine the risk of malignancy. Through this study, we found that the categories and total level of TI‐RADS were also significantly associated with LLNM. When considering the application of the model in different hospitals, the advantage of TI‐RADS lies in its widespread clinical adoption, obviating the need for additional training for radiologists. In contrast, Luo et al.'s^16^ predictive model relies on eight thyroid ultrasound image features, which may pose challenges for dissemination and adoption across diverse medical institutions.

The current exploration of risk factors for LLNM in MTC is mainly based on Machens' study. He found that basal Ctn beyond thresholds of 20, 50, 200, and 500 pg/mL, indicating LNM in ipsilateral central and lateral neck, contralateral central neck, contralateral lateral neck, and upper mediastinum, respectively.^6^ As the number of central lymphnode metastases increases, the probability and extent of lateral cervical lymphnode metastases would also increase.^5^ We tested basal Ctn, CLN status and our model in cN_0−1a_ patients. The result showed that our model had a better ability to detect occult LLNM than the other two, with *p* < 0.05. Therefore, our model can be used in clinical practice to decide whether to perform prophylactic lateral neck dissection.

Our choice of machine learning algorithm balances interpretability, stability, and efficiency. Logistic regression is suitable for medical applications and provides interpretability in clinical settings. Given the rarity of MTC cases, regularization is used to prevent overfitting and enhance generalization. Additionally, a grid search method is employed to comprehensively explore hyperparameter space and identify the optimal model. Among other commonly used modeling techniques, decision trees tend to overfit and are not suitable for high‐dimensional data, while random forest models are complex and challenging to interpret. 5‐fold cross‐validation leverages the limited MTC data by ensuring that each case is used for both training and testing, thus enhancing the model's robustness.

However, our article also has some shortcomings. On the one hand, this study only included the basal Ctn. Other tumor markers such as CEA and procalcitonin, which was regarded as potential tumor markers,^23^ were not included in the study. But in our experience, these tumor markers are also affected by many other external factors and are not superior to Ctn for the diagnosis and prognosis evaluation. In addition, since pentapeptide gastrin is not approved for use in mainland China, it is not possible to measure the stimulated Ctn. On the other hand, the biggest regret of this study is that it did not include an external validation dataset. Because for such a rare disease, it may be necessary to contact multiple hospitals to complete enough data, which is also our wish for future research.

In conclusion, by using a machine learning algorithm, we developed a predictive model for LLNM of MTC based on clinical characteristics and ultrasound images. Our model has good discrimination and can significantly improve the prediction ability of occult LLNMs in practical applications. This will further provide decision support for prophylactic LND in patients with clinically negative findings in the lateral neck.

## AUTHOR CONTRIBUTIONS

**Xiwei Zhang:** Formal analysis (supporting); software (supporting); writing – original draft (equal). **Xiaohui Zhao:** Software (supporting); writing – original draft (equal). **Lichao Jin:** Methodology (equal). **Qianqian Guo:** Methodology (equal). **Minghui Wei:** Conceptualization (supporting). **Zhengjiang Li:** Conceptualization (supporting). **Lijuan Niu:** Conceptualization (supporting); methodology (equal). **Zhiqiang Liu:** Formal analysis (lead); software (lead); writing – review and editing (equal). **Changming An:** Conceptualization (lead); writing – review and editing (equal).

## FUNDING INFORMATION

This study was supported by the National Natural Science Foundation of China (General Program) (82171965), the Sanming Project of Medicine in Shenzhen (No. SZSM201911006), the Beijing Hope Run Special Fund of Cancer Foundation of China (LC2021B01), and Special Research Fund for Central Universities, Peking Union Medical College, the CAMS Innovation Fund for Medical Sciences (CIFMS) (2022‐I2M‐C&T‐B‐075).

## Supporting information


Appendix S1.

## Data Availability

Author elects to not share data.
