# Supplementary material for: The machine learning‐based model for lateral lymph node metastasis of thyroid medullary carcinoma improved the prediction ability of occult metastasis
Source: Cancer Med. 2024 May 29;13(11):e7155. doi: 10.1002/cam4.7155 (PMC11135018; doi:10.1002/cam4.7155)

**Supplementary Note 1.** LLNM prediction model

The regularized logistic regression model for LLNM prediction was established and the probability of the LLNM positive class $P\left( y_{i}=1|X_{i} \right)$ as following:

$$\hat{p}\left( X_{i} \right)=\frac{1}{1+exp(-X_{i}\omega-\omega_{0})}, (1)$$

where is $y_{i}$ takes values in the set {0, 1} for $i$th the patient, $X_{i}$ is clinical features, and$\omega$ and $\omega_{0}$ are coefficients and intercept. The optimization process is to minimize the following cost function:

$$min C\sum_{i=1}^{n} (-y_{i}\log\left( \hat{p}\left( X_{i} \right) \right)-(1-y_{i})log(1-\hat{p}\left( X_{i} \right)))+r(\omega), (2)$$

where $r(\omega)$ is the regularization penalty term and there are four choices as presented in Supplementary Note 1. Table 1, and $C$ is the factor to adjust the inverse of regularization strength. The advantage of regularization is that it can improve stability.

The hyperparameters tuning are the important part of model training and determines the performance of the model. The hyperparameters for LLNM prediction model included optimizer, regularization penalty term ($r(\omega)$) and inverse of regularization strength ($C$). The search space of hyperparameters that need to be tuned were summarized in Supplementary Note 1. Table 2. The hyperparameters of the LLNM prediction model are optimized by cross-validated exhaustive grid-search over the search space. The strategy to select the best hyperparameters is to select the most accurate prediction in the training set.

In summary, the optimization algorithm of ‘lbfgs’ was used as optimizer. The $\mathcal{l}_{2}$ regularization term is applied as penalty. The inverse of regularization strength $C$ is set to 1. The tolerance for stopping criteria is 1e-4. The maximum number of iterations taken for the solvers to converge is 1000. The parameters of the LLNM prediction model of $\omega$ and $\omega_{0}$ were presented in Supplementary Note 1. Table 3 in 5-fold cross-validation. The LLNM prediction model was implemented in Python software (version 3.8) with scikit-learn package (version 1.1.2).

**Supplementary Note 1. Table 1**. Four choices for the regularization term $r(\omega)$ via the penalty.

| **penalty** | $\boldsymbol{r(\omega)}$ |
| --- | --- |
| $\mathcal{l}_{1}$ | ${\parallel\omega\parallel}_{1}$ |
| $\mathcal{l}_{2}$ | $\frac{1}{2}\omega^{T}\omega$ |
| ElasticNet* | $\frac{1-\rho}{2}\omega^{T}\omega+\rho{\parallel\omega\parallel}_{1}$ |
| None | 0 |

*****For ElasticNet, $\rho$ ($0\leq\rho\leq1$) controls the strength of $\mathcal{l}_{1}$ regularization versus $\mathcal{l}_{2}$ regularization. ElasticNet is equivalent to $\mathcal{l}_{1}$ when $\rho=1$ and equivalent to $\mathcal{l}_{2}$ when $\rho=$0.

**Supplementary Note 1. Table 2**. Hyperparameter optimizing in LLNM prediction model training.

| **Index** | **Hyperparameter** | **Search space** |
| --- | --- | --- |
| 1 | optimizer | newton-cg, lbfgs, liblinear, sag, saga |
| 2 | regularization term $r(\omega)$ | $\mathcal{l}_{1}$, $\mathcal{l}_{2}$, Elasticnet, None |
| 3 | regularization strength $C$ | 0.1, 1, 10,100 |

**Supplementary Note 1. Table 3**. Coefficients and intercept of logistic regression model for LLNM prediction in 5-fold cross-validation. Coefficients from **w_1_** to **w_7_** are corresponding to gender, multifocality, central lymphnode status，TI-RADS margin and level, tumor size, and basal Ctn.

|  | **fold 1** | **fold 2** | **fold 3** | **fold 4** | **fold 5** |
| --- | --- | --- | --- | --- | --- |
| **w_1_** | 0.160370853 | 0.792506051 | 0.587721768 | 0.886797821 | 0.43811746 |
| **w_2_** | 1.020248983 | 0.375574019 | 0.682811745 | 0.467382948 | 0.579286568 |
| **w_3_** | 3.234464144 | 3.534881346 | 3.483043309 | 3.19932865 | 3.124284378 |
| **w_4_** | 0.737476006 | 0.805735276 | 0.799660577 | 0.995040859 | 1.082561825 |
| **w_5_** | 0.709494885 | 0.778059389 | 0.706888586 | 0.474127004 | 0.439849811 |
| **w_6_** | 0.080656731 | 0.088556197 | 0.080724948 | 0.066709165 | 0.085806165 |
| **w_7_** | 0.000179852 | 0.00020761 | 0.000255234 | 0.000289349 | 0.000215234 |
| **w_0_** | -7.765497046 | -8.54419959 | -8.025566039 | -7.15745917 | -7.363116887 |

**Supplementary Note 2**. Quantitative Evaluation Indexes

The probability threshold for the accuracy calculation was set to 0.5, a predicted probability of larger than 0.5 was classified as LLNM positive, and other values were classified as LLNM negative. LLNM positive was correctly identified as LLNM positive, and this case was recorded as true positive (TP). LLNM negative was identified as LLNM positive, and this case was recorded as false negative (FN). LLNM positive was identified as LLNM negative, and this case was recorded as false positive (FP). LLNM negative was correctly identified as LLNM negative, and this case was recorded as true negative (TN). Then the accuracy (ACC), sensitivity (SEN) / recall (REC), specificity (SPE), positive predictive value (PPV) / precision (PRE), negative predictive value (NPV), Matthew's correlation coefficient (MCC) and F1 score (F1) can be calculated as:


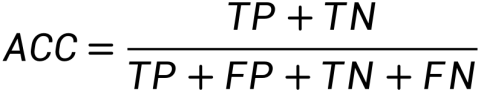


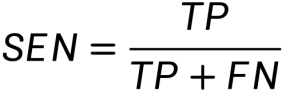


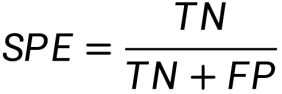


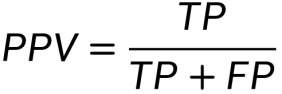


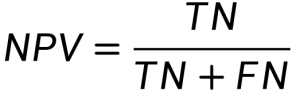


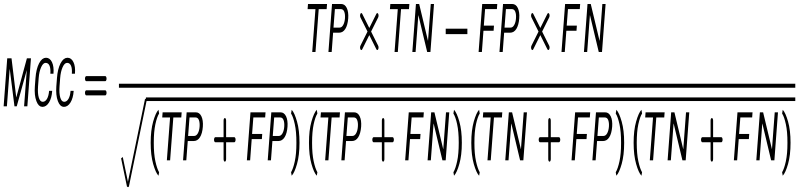


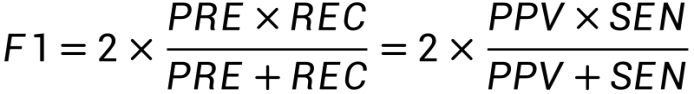


**Supplementary Note 3**. Decision curve analysis (DCA)

The decision curve analysis (DCA) was used to test the clinical usefulness of the regularized logistic regression model in LLNM prediction. The net benefit of the LLNM positive group can be calculated as following:

$$Net benifit treated= \frac{TP}{n}-\frac{FP}{n}\frac{P_{t}}{1-P_{t}}, (s2-1)$$

$$Net benifit untreated= \frac{TN}{n}-\frac{FN}{n}\frac{P_{t}}{1-P_{t}}, (s2-2)$$

$$treated-treat all= untreated\frac{P_{t}}{1-P_{t}}, (s2-3)$$

$$Net benefit treat all=\frac{TP+FN}{n}-\frac{TN+FP}{n}\frac{P_{t}}{1-P_{t}}, (s2-4)$$

where $TP$ is the number of LLNM positive patients correctly identified as LLNM positive, $FN$ is the number of LLNM negative patients identified as LLNM positive, $FP$ is the number of LLNM positive patients identified as LLNM negative, $TN$ is the number of LLNM negative patients correctly identified as LLNM negative, $n$ is the total number of patients, $P_{t}$ is the probability threshold.

Ethics approval by National GCP Center for Anticancer Drugs, The Independent Ethics Committee


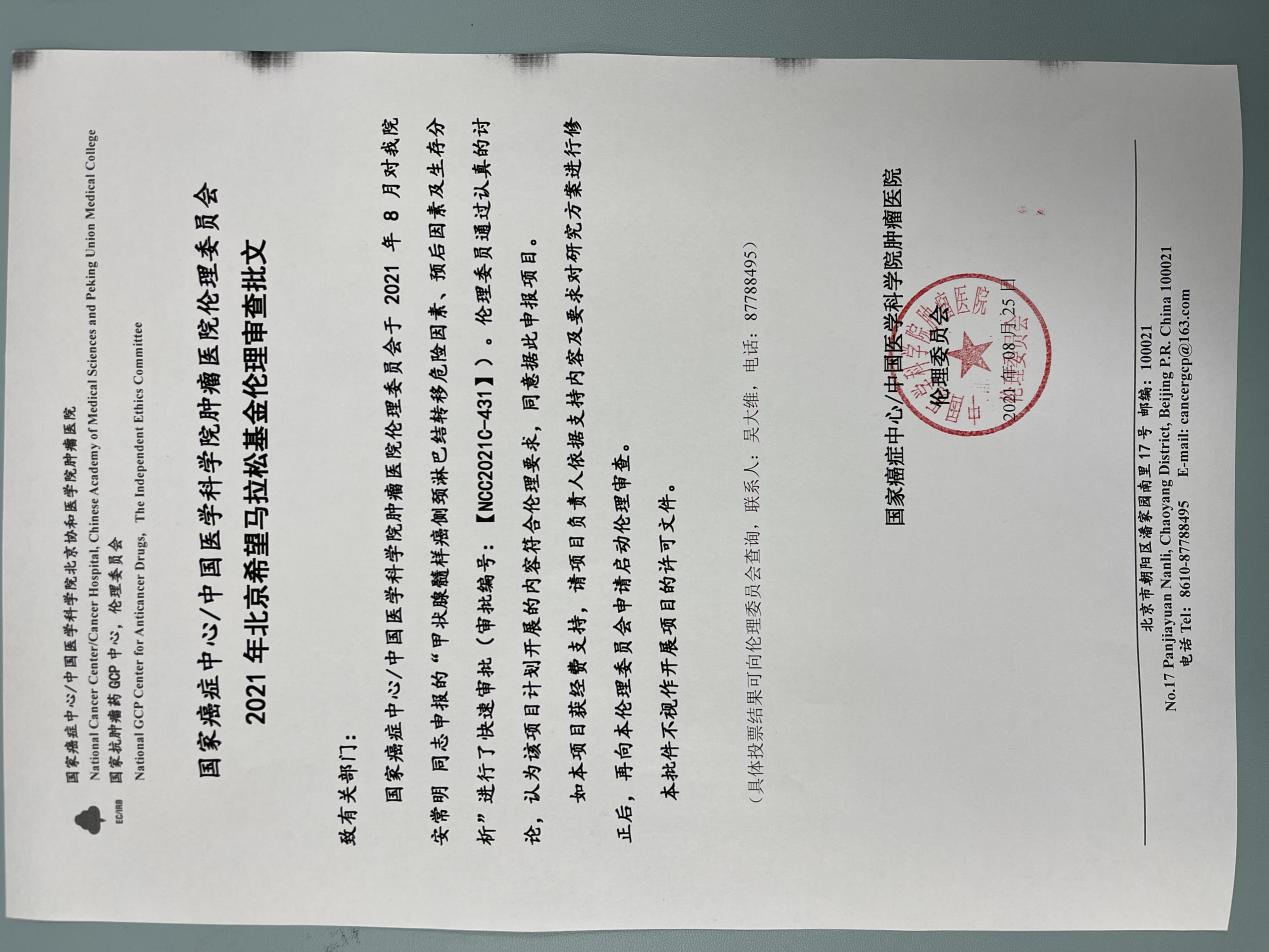

Supplement: Supplementary file 1 — Appendix S1. [file CAM4-13-e7155-s001.docx]
